# Retrospective evaluation of clear aligners' adverse effects on open gingival embrasures among orthodontic patients

**DOI:** 10.6026/973206300212984

**Published:** 2025-09-30

**Authors:** Raju B.S., Kanchan Sharma, Deepika Yadav, Keerti Kaushik, Shashank Gaikwad, Aditi Satish Lawale, Miral Mehta

**Affiliations:** 1 Department of Orthodontics and Dentofacial Orthopaedics, Maharana Pratap College of Dentistry and Research Centre, Gwalior, Madhya Pradesh, India; 2 Department of Orthodontics and Dentofacial Orthopaedics, Awadh Dental College and Hospital, Jamshedpur, Jharkhand, India; 3 Consultant Endodontist, Captain Nandlal Yadav Multi-Speciality Hospital, Rewari, Haryana, India; 4 Department of Orthodontics and Dentofacial Orthopaedics, ITS Dental College, Greater Noida, Uttar Pradesh, India; 5 Department of Orthodontics, Bharati Vidyapeeth (Deemed to be University) Dental College and hospital, Navi Mumbai, India; 6 Intern, Bharati Vidyapeeth Dental College and Hospital, Pune, India; 7 Department of Pediatric and Preventive Dentistry, Karnavati School of Dentistry, Karnavati University, Gandhinagar, Gujarat, India

**Keywords:** Clear aligners, open gingival embrasure, black triangle, orthodontic treatment, interproximal reduction, esthetic complications

## Abstract

Open gingival embrasures (OGEs), commonly called "black triangles," are a frequent esthetic and periodontal complication reported in patients undergoing clear aligner therapy. This retrospective study analyzed clinical records of 80 patients treated between 2020 and 2022 to assess the prevalence and risk factors of OGEs. Digital records and casts were evaluated for OGEs, with variables such as age, gender, treatment duration, incisor movement, interproximal reduction (IPR) and aligner compliance analyzed using chi-square and logistic regression. Data showed OGEs in 48.75% of patients, most commonly between maxillary central incisors, with significant associations to incisor proclination and lack of IPR. Thus, we show that careful planning of tooth movement and contact point management is essential to minimize OGEs in clear aligner therapy.

## Background:

Clear aligner therapy has become a widely accepted alternative to conventional fixed orthodontic appliances due to its superior esthetics, enhanced comfort and improved oral hygiene maintenance during treatment [1]. These thermoplastic appliances apply sequential forces to guide teeth into desired positions and are particularly preferred by adult patients seeking discreet orthodontic solutions [2]. Despite their benefits, recent studies have raised concerns about the unintended adverse effects of aligner therapy, including root resorption, poor torque control and open gingival embrasures (OGEs), also referred to as "black triangles" [3, 4]. OGEs are characterized by the loss of interdental papilla between anterior teeth, resulting in unaesthetic spaces that can negatively impact smile appearance and patient satisfaction [5]. This condition not only poses an esthetic challenge but also predisposes patients to food impaction and periodontal issues due to the loss of natural protection provided by gingival tissue [6]. Factors contributing to OGEs during orthodontic treatment include crown morphology, root angulation, tooth movement, periodontal biotype and the application of interproximal reduction (IPR) [7]. In clear aligner therapy, precise control of tooth movement is essential to prevent divergence of roots and excessive flaring, both of which are known to reduce papillary height and create OGEs [8]. Moreover, unlike fixed appliances, aligners do not produce continuous forces and often require high levels of patient compliance, which can indirectly influence treatment outcomes including the integrity of interdental papilla [9]. However, literature evaluating the frequency and risk factors of OGEs specifically in patients treated with clear aligners remains limited. One of the primary etiological mechanisms behind open gingival embrasures in clear aligner therapy is the excessive proclination or uncontrolled tipping of anterior teeth, which results in the displacement of contact points apically and divergence of roots [3]. The reduced overlap between proximal surfaces leaves inadequate space for the interdental papilla to occupy, thereby leading to the appearance of a black triangle. Unlike fixed appliances, which allow three-dimensional bracket control, aligners rely on plastic stiffness and programmed tooth movement, often making torque control more difficult, particularly in the anterior region [4]. Another significant factor influencing the development of OGEs is the absence or inadequate execution of interproximal reduction (IPR). IPR is designed to reshape the mesiodistal dimension of teeth to create more favorable contact areas and reduce crowding without flaring incisors [5]. Studies suggest that proper application of IPR can move the contact point more apically, allowing better papillary fill and minimizing the risk of OGEs [6]. In clear aligner protocols, when IPR is omitted or performed late, the natural divergence caused by tooth movement becomes more pronounced, thus increasing the likelihood of interdental papilla loss. Patient-related factors such as age and gingival biotype also play a role in the presence and severity of OGEs. Older patients often have reduced gingival elasticity and compromised vascularity, which limits the ability of the papilla to regenerate following orthodontic movement [7]. Additionally, patients with thin biotypes are more prone to recession and papillary shrinkage compared to those with thick biotypes, especially in the anterior maxilla. While aligner therapy is less invasive, the prolonged force application and poor adaptation in areas with anatomical undercuts can further compromise soft tissue health [8]. Therefore, early identification of patients at risk and implementation of preventive strategies during planning is crucial for achieving both functional and esthetic outcomes [9]. Therefore, it is of interest to assess the prevalence of OGEs in orthodontic patients treated with clear aligners and to investigate the association between various treatment-related factors and the development of OGEs, thereby guiding clinicians in identifying potential risk factors during treatment planning and execution.

## Materials and Methods:

The digital records of 80 patients who completed clear aligner therapy between January 2020 and December 2022 were evaluated. Inclusion criteria involved patients aged 18-35 years with completed non-extraction orthodontic treatment using clear aligners (Invisalign® or equivalent), with available pre-treatment and post-treatment intraoral photographs and digital study models. Patients with previous periodontal disease, fixed appliance history, or incomplete records were excluded. Digital intraoral photographs and STL files of dental casts were analyzed using standardized protocols. The presence of open gingival embrasures (OGEs) was assessed between maxillary and mandibular anterior teeth (canine to canine). OGEs were recorded when a visible triangular space was present between the gingival papilla and the contact point in post-treatment images, as judged independently by two calibrated orthodontists. In cases of disagreement, a third examiner evaluated the image to reach consensus. Patient-related variables (age, gender) and treatment-related parameters such as treatment duration, amount of incisor proclination (measured via cephalometric analysis), interproximal reduction (IPR) and number of aligners used were recorded. The degree of anterior tooth movement was classified based on ClinCheck® or equivalent software records. Aligners' wear compliance was noted from follow-up documentation and patient reports. Descriptive statistics were computed and the prevalence of OGEs was expressed as a percentage. Associations between OGEs and potential contributing factors were assessed using chi-square test and binary logistic regression. A p-value of <0.05 was considered statistically significant. All data analyses were performed using SPSS version 26.0 (IBM Corp., Armonk, NY, USA).

## Results:

A total of 80 patients (38 males and 42 females) who completed clear aligner therapy were included in the study. The mean age of the sample was 26.7 ± 4.2 years. Open gingival embrasures (OGEs) were observed in 39 patients, corresponding to a prevalence of 48.8%. Regarding arch distribution, OGEs were more frequent in the maxillary arch (31 cases) compared to the mandibular arch (24 cases). The most commonly affected site was the maxillary central incisor region (25 cases; 62.0%), followed by the mandibular central incisor region (20 cases; 51.3%). Less frequently, OGEs occurred in the maxillary lateral-canine area (6 cases; 15.4%) and the mandibular lateral-canine area (4 cases; 10.3%) (Table 1 - see PDF). When stratified by age, OGEs were present in 17 patients below 25 years (45.8%) and in 22 patients aged 25 years or older (52.6%). By gender, OGEs were observed in 18 males (47.4%) and 21 females (50.0%). Statistical analysis revealed no significant association between OGEs and either age group (p = 0.52) or gender (p = 0.77) (Table 2 - see PDF). A significant correlation was found between the degree of incisor proclination and the occurrence of OGEs. Patients showing proclination >10° had a higher prevalence (71.4%) of OGEs compared to those with proclination ≤10° (34.6%). Similarly, the absence of interproximal reduction (IPR) was associated with an increased frequency of OGEs (p = 0.04) (Table 3 - see PDF). Treatment duration and aligner wear compliance were also evaluated. Patients undergoing treatment for more than 18 months had a slightly higher occurrence of OGEs (55%) compared to those treated in ≤18 months (42.1%). Poor compliance with aligner wear was associated with an increased likelihood of OGEs (p = 0.02) (Table 4 - see PDF). These findings suggest that incisor proclination, IPR omission and poor aligner compliance are significantly associated with the development of open gingival embrasures during clear aligner therapy.

## Discussion:

Open gingival embrasures (OGEs), commonly referred to as "black triangles," are a frequent esthetic and functional complication observed in patients following orthodontic treatment, particularly with clear aligner therapy. In the present retrospective study, OGEs were seen in 48.75% of the patients treated with clear aligners, predominantly in the maxillary anterior region. This aligns with prior literature where the incidence of OGEs ranged from 38% to 58% following orthodontic treatment using removable appliances [1, 2]. The pathogenesis of OGEs is multifactorial, involving alterations in tooth positioning, gingival architecture and the interproximal contact relationship. One major factor identified in this study was the degree of incisor proclination, with significant proclination (>10°) associated with a higher prevalence of OGEs (p = 0.03). This finding corroborates earlier studies indicating that labial movement of anterior teeth increases the distance from the contact point to the alveolar crest, which negatively influences papilla height [3, 4]. Excessive flaring leads to the root divergence and reduces the space for papilla fill, thereby contributing to the black triangle effect [5]. Another critical variable was the absence of interproximal reduction (IPR), which showed a significant correlation with the occurrence of OGEs. Properly executed IPR not only prevents excessive proclination by creating space but also helps in reshaping the contact areas between teeth, potentially lowering the contact point and promoting gingival fill [6, 7]. In aligner therapy, delayed or insufficient IPR can lead to uncontrolled spacing and contact loss, both of which predispose to papillary loss [8]. Aligner wear compliance also emerged as a notable factor, where poor compliance was significantly associated with OGEs (p = 0.02). This could be attributed to inconsistent force application leading to non-ideal tooth movement patterns and compromised root control [9]. Unlike fixed appliances, which exert continuous forces, aligners rely heavily on patient adherence for optimal results. Missed wear time can disrupt the precision of programmed movements, ultimately affecting soft tissue adaptation and stability [10]. Age and gender did not show a statistically significant association with OGEs in the current study, which is consistent with some previous investigations [11]. However, other studies have suggested that older individuals are more prone to papillary loss due to reduced tissue elasticity and vascularity [12]. Although our study observed slightly higher OGE incidence in patients over 25 years, the difference was not statistically meaningful. The influence of gingival biotype was not directly assessed but remains a crucial determinant of soft tissue response to orthodontic forces [13]. Furthermore, the study highlighted that prolonged treatment duration (>18 months) might contribute to a marginally increased risk of OGEs. Extended orthodontic therapy may cause chronic low-grade inflammation or gingival remodeling that adversely impacts papilla fills [14]. Therefore, clinicians must aim for efficient treatment timelines while maintaining optimal biomechanics. While clear aligner systems have evolved with integrated features like optimized attachments and precision staging to manage complex tooth movements, their ability to control torque and root angulation in the anterior region remains inferior to fixed appliances [15]. These limitations demand a thorough evaluation of patient-specific risk factors and appropriate intervention strategies, such as early detection of embrasure formation, careful staging of IPR and use of overcorrection movements when needed.

## Conclusion:

Open gingival embrasures are a prevalent esthetic concern in patients treated with clear aligners, particularly in cases with significant incisor proclination and inadequate IPR. Patient compliance and proper treatment planning play crucial roles in minimizing this complication. Early identification and preventive strategies can enhance both periodontal health and post-treatment esthetics.
